# Type III CD38 is present in the membrane of neurosecretory vesicles and has a cytosol‐facing catalytic domain in primate oxytocin neurons

**DOI:** 10.1111/jne.70187

**Published:** 2026-04-19

**Authors:** Tatsuki Miyamoto, Akari Matsushima, Akito Otubo, Chihong Song, Kazuyoshi Murata, Takumi Oti, Hirotaka Sakamoto

**Affiliations:** ^1^ Department of Biology, Faculty of Environmental, Life, Natural Science and Technology Okayama University Okayama Japan; ^2^ Department of Biology, Faculty of Science Okayama University Okayama Japan; ^3^ Exploratory Research Center on Life and Living Systems (ExCELLS) National Institutes of Natural Sciences Okazaki Aichi Japan; ^4^ National Institute for Physiological Sciences National Institutes of Natural Sciences Okazaki Aichi Japan; ^5^ Department of Physiological Sciences, School of Life Science The Graduate University for Advanced Studies (SOKENDAI) Okazaki Aichi Japan; ^6^ Department of Convergence Medicine, School of Medicine Pusan National University Yangsan Republic of Korea

**Keywords:** CD38, cyclic ADP‐ribose, membrane topology, neurosecretory vesicles, oxytocin

## Abstract

CD38, an ADP‐ribosyl cyclase that generates cyclic ADP‐ribose (cADPR), is essential for Ca^2+^‐dependent oxytocin release. However, its subcellular localisation and membrane topology within oxytocin neurones have remained unclear. We investigated the distribution and orientation of CD38 in oxytocin‐producing neurones of Japanese macaques (*Macaca fuscata*) using immunoelectron microscopy combined with biochemical isolation of neurosecretory vesicles (NSVs). CD38 immunoreactivity was selectively detected on oxytocin‐containing NSVs in axon terminals in the posterior pituitary and dendrites of the supraoptic nucleus, whereas vasopressin vesicles and the plasma membrane lacked detectable labelling. Cryo‐electron microscopy confirmed the structural integrity of purified NSV fractions, and Western blotting verified the presence of CD38 protein within these fractions. Permeabilisation‐dependent immunogold labelling further demonstrated that the NSV membrane localisation of CD38 and that the N‐terminal region of CD38 is oriented toward the vesicle lumen, consistent with a type III membrane topology in which the catalytic domain faces the cytosol. This arrangement positions the active site near cytosolic NAD^+^, enabling localised cADPR production adjacent to Ca^2+^‐mobilising channels that support regulated exocytosis. These findings identify, in primate oxytocin neurones, a previously unrecognised, vesicle‐associated pool of CD38 with a cytosol‐facing catalytic domain and provide a structural framework for understanding how intracellular type III CD38 contributes to neuropeptide release.

## 1 INTRODUCTION

CD38 is a single‐transmembrane protein classified as a cell surface antigen that is highly expressed in both hematopoietic cells and the brain. This multifunctional enzyme possesses ADP‐ribosyl cyclase activity, catalysing the synthesis of cyclic ADP‐ribose (cADPR) from nicotinamide adenine dinucleotide (NAD^+^) as a substrate, and is predominantly localised to the plasma membrane.^1^, ^2^, ^3^ In addition to its cyclase activity, CD38 exhibits NAD^+^ glycohydrolase activity, enabling its participation in multiple signalling pathways. The enzymatic product cADPR serves as a critical second messenger that triggers intracellular Ca^2+^ mobilisation through ryanodine receptors on the endoplasmic reticulum, thereby regulating diverse cellular responses, including neurotransmitter release, hormone secretion, and cellular metabolism.^4^, ^5^, ^6^, ^7^

Oxytocin is a neuropeptide hormone known for its essential role in social bonding, affiliative behaviour, and reproduction.^8^, ^9^ Mice deficient in the CD38 gene exhibit specific impairment in the secretion of oxytocin and display markedly attenuated maternal behaviour.^10^ Despite normal oxytocin synthesis in hypothalamic neurones, the CD38 knockout mice fail to release adequate amounts of oxytocin into the bloodstream in response to physiological stimuli.^10^ These findings established CD38 as a key enzyme for oxytocin release; however, the detailed molecular mechanisms underlying this specific regulatory role remain unclear.

A critical unresolved question concerns the subcellular localisation and membrane topology of CD38 in oxytocin neurones. While CD38 is predominantly described as a plasma membrane protein, its role in regulated peptide hormone release suggests a potential association with intracellular compartments, particularly neurosecretory vesicles (NSVs). The membrane topology of CD38 on these vesicles is of fundamental importance because the orientation of its catalytic domain determines NAD^+^ substrate accessibility and cADPR product delivery to the appropriate cellular compartments. Furthermore, dendritic oxytocin release can occur independently of action potentials,^11^ suggesting the possibility of compartment‐specific regulatory mechanisms that may involve distinct CD38 localisation patterns.

Here, we investigate the molecular mechanisms underlying CD38‐mediated oxytocin release, with particular emphasis on its subcellular localisation and membrane topology. Japanese macaque monkeys (*Macaca fuscata*) were used as a primate model. Employing immunoelectron microscopy, we examined the precise subcellular distribution and membrane orientation of CD38 in oxytocin neurones to elucidate how this enzyme couples NAD^+^ metabolism to regulated oxytocin release. Our findings in a non‐human primate model provide insights relevant to human neuroendocrine physiology and may inform therapeutic strategies for disorders involving social cognitive deficits, including autism spectrum disorders.

## 2 MATERIALS AND METHODS

### 2.1 Animals

Three male (2–9‐year‐old, weight 2.4–12.6 kg) and four female (9–11‐year‐old, weight 7.2–8.2 kg) monkeys were used in this study. Monkeys were maintained in a temperature‐controlled (22–24°C) room under a daily photoperiod of 12:12‐h light/dark cycle (lights off at 8:00 p.m.). These animals were checked and shown to be free of specific pathogens. Food and water were available ad libitum. All animals were kept in individual cages. The housing and experimental protocols adhered to the guidelines of the Ministry of Education, Culture, Sports, Science and Technology (MEXT) of Japan and were in accordance with the Guide for the Care and Use of Laboratory Animals prepared by Okayama University (Okayama, Japan). All efforts were made to minimise animal suffering and reduce the number of animals used in this study. For immunoelectron microscopy, animals were perfused with fixative, whereas tissues used for NSV isolation and Western blotting were snap‐frozen without fixation; the two sample sets were entirely independent with no overlap between groups. Samples for Western blotting and NSV isolation were processed independently from each frozen sample.

### 2.2 Experimental procedures

#### 2.2.1 Tissue processing

Monkeys (*n* = 2 females; *n* = 2 males) were deeply anaesthetised with an overdose of sodium pentobarbital (50–90 mg/kg body weight) and transcardially perfused with physiological saline. The posterior pituitaries were dissected and quickly frozen. Following the saline perfusion, several monkeys were perfused with 4% formaldehyde in 0.1 M phosphate buffer (PB; pH 7.4). Brains and pituitaries were immediately removed and post‐fixed overnight at 4°C in the same fixative.

#### 2.2.2 Isolation of NSVs

Isolation of NSVs from posterior pituitaries was performed according to the method developed for exosome isolation^12^ with slight modifications, because the sizes of exosomes and NSVs are similar. In brief, posterior pituitary preparations (*n* = 2 females; *n* = 1 male) were homogenised using BioMasher I (Nippi, Tokyo, Japan) and suspended in PBS. Homogenates were first filtered through a Millex‐GP (0.22 μm) filter (Merck Millipore, Darmstadt, Germany), then transferred to 0.5 mL Amicon Ultrafiltration tubes with a 10 kDa membrane (Merck Millipore). These ultrafiltration tubes were centrifuged at 12,000×*g* for 30 min at 4°C. Subsequently, the NSV fractions were isolated by ultrafiltration.

#### 2.2.3 Western immunoblotting

Posterior pituitaries (*n* = 2 females; *n* = 1 male) and NSV fractions were subjected to Western analysis as described previously.^13^ The preparations from female monkeys were boiled in 10 μL sample buffer containing 62.5 mM tris(hydroxymethyl)‐aminomethane‐HCl (Tris–HCl; pH. 6.8), 2% SDS, 25% glycerol, 10% 2‐mercaptoethanol, and a small amount of bromophenol blue. These samples were run on a 4%–20% SDS‐PAGE and electroblotted onto a polyvinylidene difluoride membrane (Bio‐Rad Laboratories, Hercules, CA, USA) using a semidry blotting apparatus (Bio‐Rad Laboratories). The blotted membranes were blocked with PVDF blocking reagent (TOYOBO, Tokyo, Japan) for 30 min at room temperature and incubated overnight at 4°C with rabbit polyclonal antibodies against human CD38 (Abiocode, Cat# R0343‐1, Lot# 2342, Agoura Hills, CA, USA)^14^ 1:10,000 dilution in Can Get Signal Solution 1 (TOYOBO). The blotted membranes were washed three times with 0.05% Tween 20 in 50 mM Tris‐buffered saline (TBST; pH 7.6) and incubated with horseradish peroxidase‐conjugated goat polyclonal antibody against rabbit IgG (Bio‐Rad Laboratories) 1:100,000 dilution in Can Get Signal Solution 2 (TOYOBO) for 1 h at room temperature. After washing three times with TBST, blots were visualised by Clarity Western ECL Substrate (Bio‐Rad Laboratories). Western analyses were independently repeated at least three times using different samples and all gave similar results.

#### 2.2.4 Immunoelectron microscopy

Brains were cut into 50‐μm‐thick coronal sections with a Linear‐Slicer (PRO 10; Dosaka EM, Kyoto, Japan). The brain sections and posterior pituitaries (*n* = 2 females; *n* = 2 males) were rinsed several times with 0.1 M PB for 5 min each, then dehydrated through increasing concentrations of methanol, embedded in LR Gold resin (Electron Microscopy Sciences, Hatfield, PA, USA), and polymerised under UV lamps at −20°C for 24 h. Ultrathin sections (70 nm in thickness) were collected on nickel grids coated with a collodion film, rinsed with PBS (pH 7.4) several times, and then incubated with 1% normal goat serum and 2% BSA in 50 mM TBS (pH 7.6) for 30 min to block non‐specific binding. Double immunoelectron microscopy was performed according to our previously described methods.^13^ The sections were incubated with a 1:50 dilution of rabbit polyclonal antibodies against CD38 and a 1:100 dilution of oxytocin antibody (4G11, Merk Millipore; mouse monoclonal)^15^ for 1 h at room temperature. The oxytocin antibody has previously been shown to be specific at the ultrastructural level.^16^ After incubation with the primary antibodies, the sections were washed with PBS and then incubated with a 1:50 dilution of a goat antibody against rabbit IgG conjugated to 5 nm gold particles (EM.GAR5; BBI Solutions, Cardiff, UK) and a goat antibody against mouse IgG conjugated to 15 nm gold particles (EM.GMHL15; BBI Solutions) for 1 h at room temperature.

Triple immunoelectron microscopy with antibodies against CD38, vasopressin‐associated neurophysin II (NPII, PS41),^17^, ^18^ and oxytocin was performed by using the front and back of ultrathin sections mounted on nickel grids without a supporting film. First, immunocytochemistry with a pair of primary antibodies (CD38 and NPII) was performed on one side of the section, and the reaction was detected using 15 nm (mouse) and 10 nm (rabbit) colloidal gold particles (BBI Solutions), respectively. Next, immunocytochemistry with the other primary mouse anti‐oxytocin antibody was performed on the opposite side of the section and detected with 5 nm colloidal gold particles (BBI Solutions). Immunoelectron microscopy studies were repeated at least three times independently using different macaques and consistently yielded similar results. Finally, the sections were contrasted with uranyl acetate and lead citrate and viewed using an H‐7650 electron microscope (Hitachi, Tokyo, Japan) operated at 80 kV. Immunocytochemical studies were repeated at least three times independently using different males, with similar results.

The prepared NSV fraction was placed on carbon‐coated nickel grids (200 mesh; Agar Scientific, Essex, UK) supported with Collodion film and incubated at RT for 2 min to allow adsorption of the NSVs onto the grids. The grids bearing adsorbed NSVs were rinsed with PBS, then blocked in 50 mM Tris‐buffered saline (TBS, pH 8.2) containing 2% BSA and 0.05% NaN3 at RT for 20 min. The grids were then incubated with rabbit anti‐human CD38 (N‐terminal) antibody (1:50) diluted in TBS for 1 h. The effect of detergent treatment (0.2% Tween 20) on the labelling pattern was also examined. After washing with PBS, the grids were incubated for 1 h at RT with a colloidal gold‐conjugated secondary antibody (anti‐rabbit IgG, 10 nm, 1:50; Abcam, ab30812, Cambridge, UK) in TBS. After washing with PBS and distilled water, the grids were negatively stained with EMstainer™ for several minutes and air‐dried. Observations were conducted using a transmission electron microscope. It should be noted that the use of primary and secondary IgG antibodies (each approximately 10 nm in size) introduces a maximum displacement of approximately 20 nm between the gold particle and the target epitope. This spatial offset does not affect the interpretation of membrane localisation in the present study.

#### 2.2.5 Cryo‐electron microscopy

The NSV fraction was observed by cryo‐electron microscopy. Three microliters of the sample aliquot was applied onto a glow‐discharged Quantifoil Mo grid R2/1 (Quantifoil MicroTools GmbH, Jena, Germany) and plunged‐frozen in liquid ethane using a Vitrobot Mark IV (Thermo Fisher Scientific, Waltham, MA, USA) at 4°C and 95% humidity. The frozen grid mounted on a 626 side‐entry cryo‐specimen holder (Gatan Inc., Pleasanton, CA, USA) was loaded into a 200 kV electron microscope equipped with an Omega‐type energy filter, JEM‐2200FS (JEOL Inc., Tokyo, Japan), and imaged at a suitable magnification on a direct electron detector, DE‐20 (Direct Electron LP, San Diego, CA, USA) using a low‐dose system.

#### 2.2.6 Statistics

Gold particle counts were compared between groups using the Mann–Whitney *U* test, with statistical significance set at *p* < 0.05.

## 3 RESULTS

**FIGURE 1:**
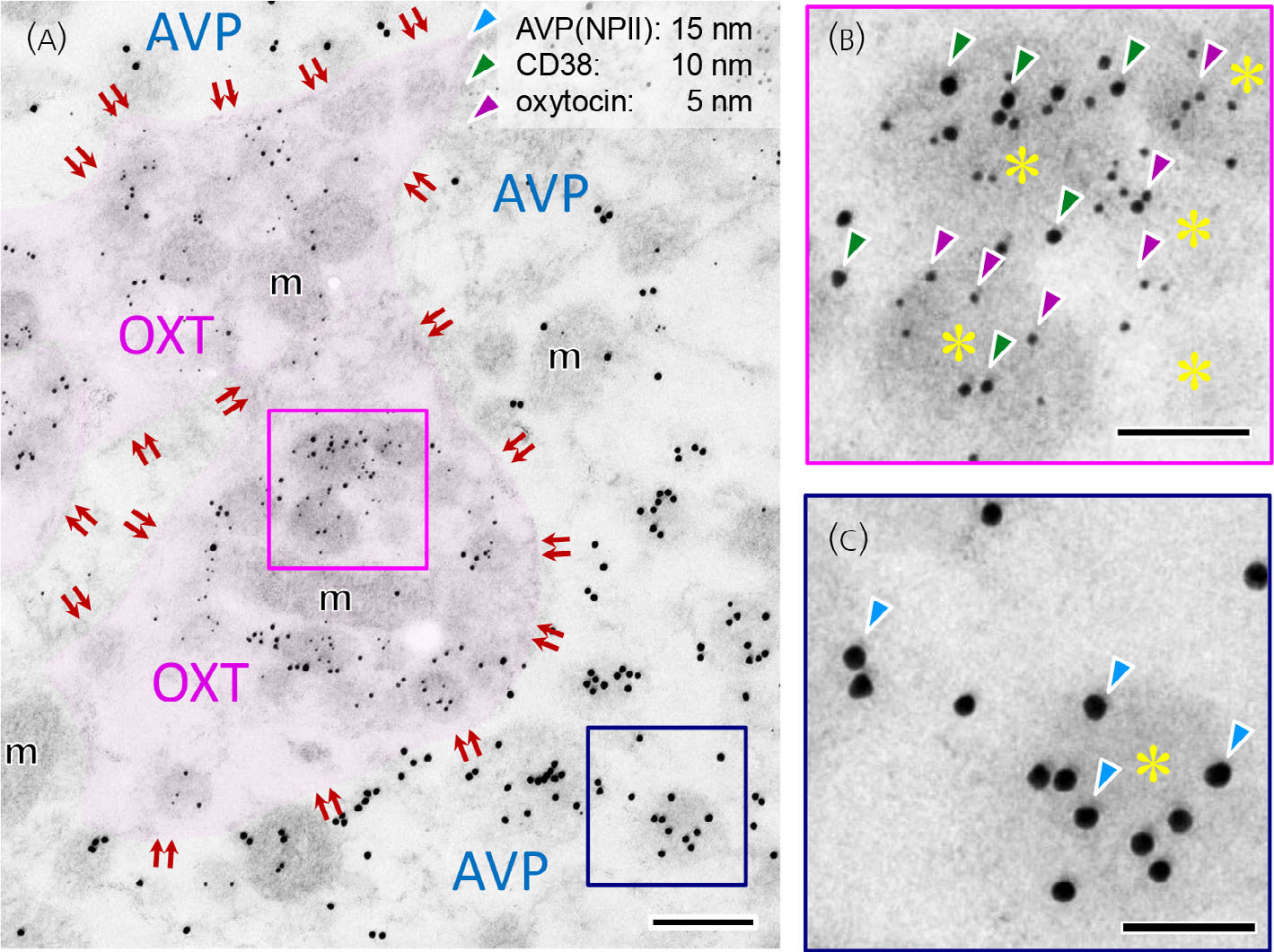
Triple immuno‐stained electron microscopy for CD38, oxytocin (OXT), and vasopressin (AVP)‐neurophysin II (NPII) in the macaque posterior pituitary gland. (a) CD38 (signalled by 10‐nm gold particles; green arrowheads) and OXT (by 5‐nm gold particles; magenta arrowheads) are both detected within the terminals of magnocellular neurosecretory axons. The OXT‐positive axonal varicosities were highlighted in magenta. Mitochondria were identified based on the presence of a double membrane and cristae. (b) The enlarged image shows that CD38‐ and OXT‐immunoreactivity colocalises with neurosecretory vesicles within the same varicosity. (c) In contrast, no CD38‐immunoreactivity is detected in neighbouring AVP (NPII) (signalled by 15‐nm gold particles; blue arrowheads) terminals. *Double arrows* indicate the plasma membrane. *Asterisks* mark neurosecretory vesicles. Scale bars, 200 nm in (a), and 100 nm in enlarged images (b and c). m, mitochondrion.

**FIGURE 2:**
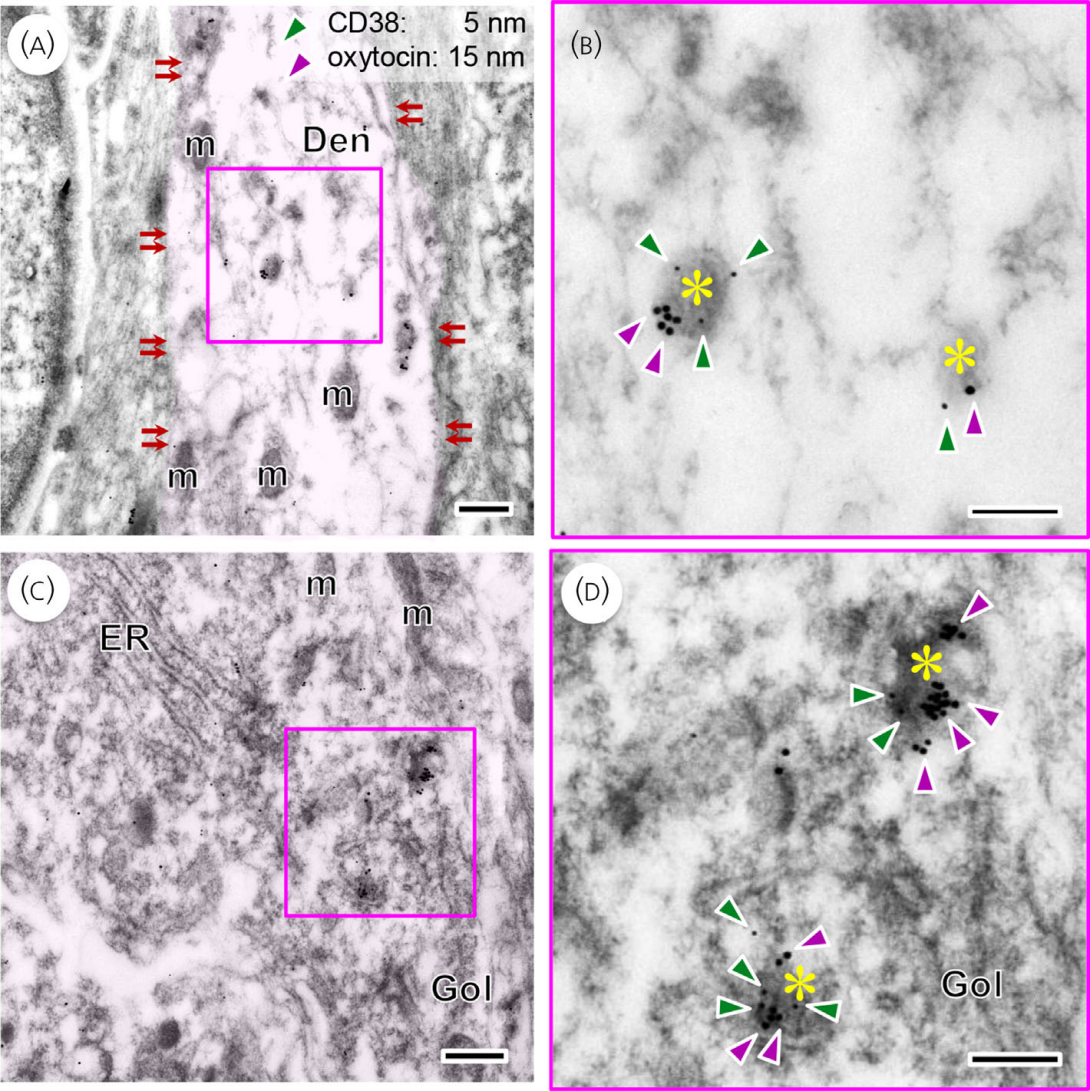
Double immuno‐stained electron microscopy for CD38 and oxytocin in the supraoptic nucleus (SON) neurones of the macaque monkey. Some neurosecretory vesicles located in the dendrites (Den) in (A) and cell bodies of SON neurones in (C) are doubly immunoreactive for CD38 (signalled by 5‐nm gold particles; green arrowheads) and oxytocin (signalled by 15‐nm gold particles; magenta arrowheads). The dendrite and soma areas were highlighted in magenta. The outlined areas in (A) and (C) are enlarged in (B) and (D), respectively. *Double arrows* indicate the plasma membrane. Asterisks mark neurosecretory vesicles. Scale bars, 500 nm in (A) and (C), 200 nm in (B) and (D). Gol, Golgi apparatus; m, mitochondrion.

The cellular localisation of CD38 in the neurohypophyseal axon terminals of oxytocin neurones was examined using immunoelectron microscopy. CD38 protein was expressed by the membranes of NSVs containing oxytocin, but not in NSVs containing vasopressin (Figure 1). In contrast, CD38 immunoreactivity was not observed on the plasma membrane (Figure 1; indicated by double arrows). Subsequently, a detailed analysis was performed on supraoptic nucleus (SON) neurones at the dendritic and soma levels. Double immunoreactivity for CD38 and oxytocin was observed in NSVs within dendrites (Figure 2), suggesting that membrane organisation may influence apparent CD38 immunoreactivity. Moreover, similar CD38‐immunoreactivity was detected in NSVs containing oxytocin distributed near the Golgi apparatus and endoplasmic reticulum (Figure 2). Again, CD38 immunoreactivity was not observed on the plasma membrane (Figure 2).

**FIGURE 3:**
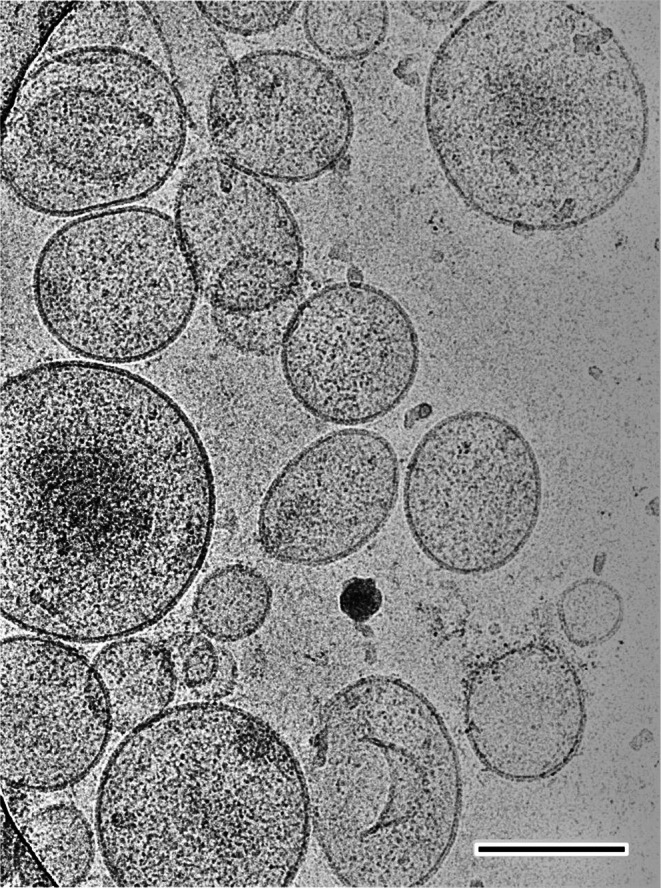
Cryo‐electron micrograph of the isolated neurosecretory vesicle fraction from the neural lobe of the posterior pituitary gland. Numerous intact neurosecretory vesicles enclosed by a lipid bilayer are observed. The mean diameter of neurosecretory vesicles in the Japanese macaque posterior pituitary has been reported to be approximately 200 nm^19^; variation in apparent profile size is attributable to differences in the plane of sectioning. Scale bar, 100 nm.

**FIGURE 4:**
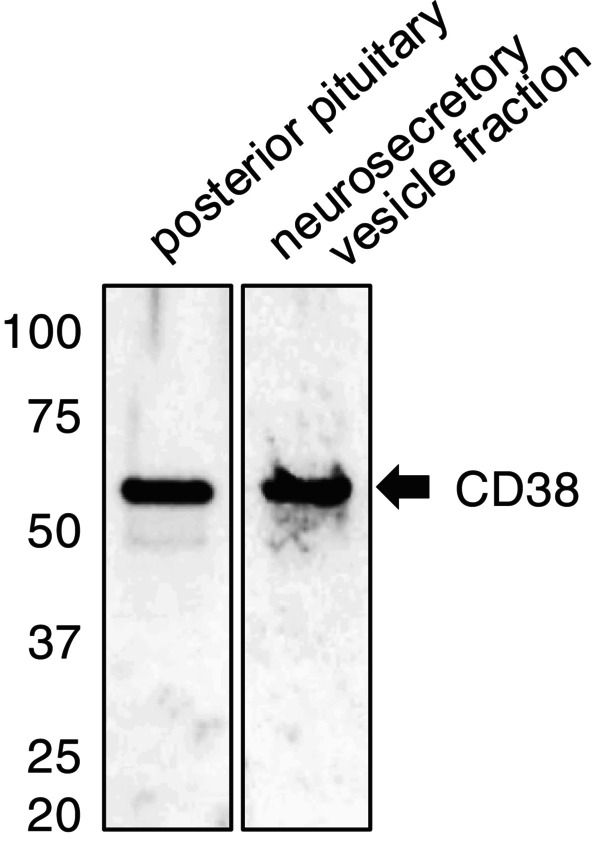
Western blot analysis of CD38. Molecular weight markers (kDa) are indicated on the left. Posterior pituitary gland (left lane) and isolated neurosecretory vesicle fraction (right lane) of the macaque show a single CD38‐positive band at approximately 54 kDa.

Next, an NSV fraction was isolated from the posterior pituitary using ultrafiltration and performed morphological analysis using cryo‐electron microscopy. Numerous intact NSVs each surrounded by a lipid bilayer were observed (Figure 3), demonstrating the high precision of the fractionation technique. These results indicate that ultrafiltration efficiently enriched oxytocin‐containing vesicles in the high‐density NSV fraction. While few vesicles were detected in the flow‐through fraction, Western blot analysis demonstrated a clear CD38‐positive band in the NSV fraction, which was comparable to that observed in the posterior pituitary gland homogenates (Figure 4). Western blot analysis for neurophysin, a well‐established marker of NSVs, also demonstrated predominant detection in the NSV fraction in a rat model system, confirming CD38 enrichment in this compartment (data not shown).

**FIGURE 5:**
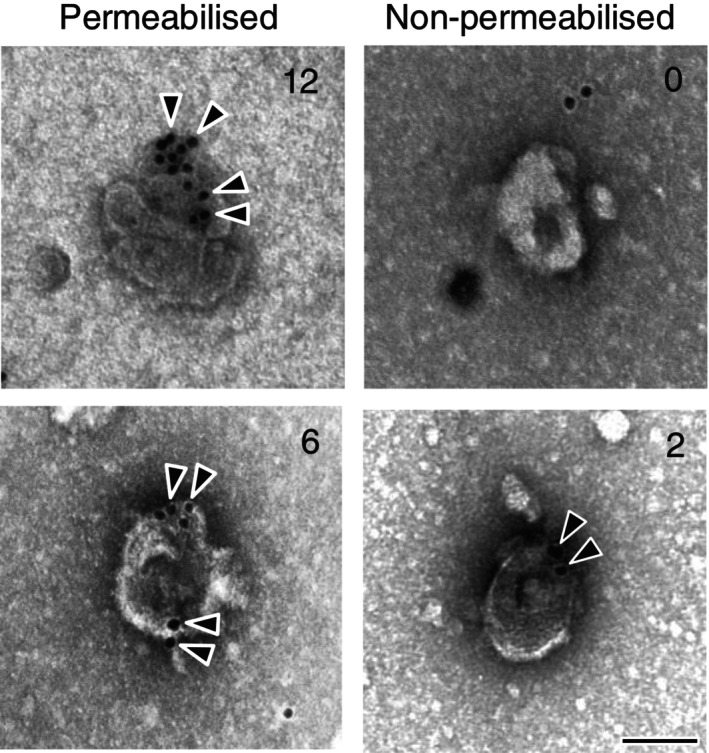
Immunoelectron microscopy of CD38 (with an antibody against the N‐terminal region) in neurosecretory vesicle fractions from the macaque posterior pituitary gland. Representative images of neurosecretory vesicles immunolabelled with or without detergent permeabilisation are shown. Arrowheads indicate gold particles, and numbers indicate the count per vesicle. A total of 106 vesicles (non‐permeabilised) and 85 vesicles (permeabilised) were analysed. Scale bar, 100 nm.

To further elucidate the membrane topology of CD38, the NSV fraction was applied to collodion/carbon‐coated EM grids, and the antibody labelling pattern was evaluated using immunoelectron microscopy. For these analyses, an antibody against the N‐terminus of CD38 was used. Under detergent‐free conditions, only epitopes exposed on the outer surface of the vesicles were labelled (Figure 5). In contrast, detergent treatment partially permeabilised the vesicle membrane, allowing antibody access to N‐terminal epitopes on the luminal side, resulting in an increased number of gold particles (Figure 5). Quantitative analysis revealed significantly more gold particles per NSV in the permeabilised group than in the non‐permeabilised group.

## 4 DISCUSSION

The following discussion presents a proposed model based on the structural and biochemical data obtained in this study; functional validation of these interpretations requires future investigation. Our findings propose that CD38 is expressed on the membrane of NSVs and adopts a membrane topology in which its N‐terminus is localised within the vesicle lumen of oxytocin‐containing NSVs (with its C‐terminus facing the cytoplasm). The membrane topology of CD38 revealed in this study provides a novel perspective on the molecular mechanism of oxytocin release. Immunoelectron microscopy showed that CD38 on the NSV membrane adopts a specific orientation with its catalytic domain facing the cytoplasm, enabling it to incorporate NAD^+^ as a substrate from the cytosolic compartment and release cADPR into the cytoplasm. This strategically positioned enzymatic activity generates cADPR in close proximity to intracellular targets, specifically ryanodine receptors on the endoplasmic reticulum and potentially two‐pore channels on lysosomes near NSVs.^16^ The locally produced cADPR may mobilise Ca^2+^ from these intracellular stores, inducing a highly localised and temporally precise increase in Ca^2+^ concentration in the immediate microenvironment surrounding NSVs. These spatially restricted Ca^2+^ microdomains are ideally positioned to facilitate Ca^2+^‐dependent fusion of NSV membranes with the plasma membrane, triggering oxytocin exocytosis. The spatial organisation of this signalling cascade, in which CD38 is present on the NSV membranes rather than extracellularly, ensures efficient coupling between cADPR production and NSV fusion, suggesting a mechanistic explanation for CD38's essential role in oxytocin release.

The membrane topology we observed suggests a functionally distinct configuration of CD38. In immune cells and other peripheral tissues, CD38 predominantly adopts a type II orientation, with its enzymatically active catalytic domain facing the extracellular space.^1^ Based on sequence analyses, the possible existence of type III CD38, with its catalytic domain facing the cytosol, was first proposed.^4^, ^20^ In contrast to the topology exhibited by a type II orientation, CD38 localised in the NSV membranes identified in this study exhibits a type III topology. Considered in the context of lipid bilayer orientation, type III CD38 similarly positions its active site toward the exterior surface of the NSV membrane. However, from a whole‐cell perspective, this configuration orients the enzymatic active site toward the cytoplasmic compartment rather than the extracellular space. This fundamental difference in topology may have profound functional implications for substrate availability and product destination.

Despite these distinct membrane orientations, type II and type III CD38 originate from the identical CD38 gene and represent the same protein without any differences in primary amino acid sequence.^21^ The divergent membrane insertion patterns are precisely regulated by multiple interconnected factors operating during protein biosynthesis and trafficking. These regulatory mechanisms may include (1) the number and distribution of positively charged amino acid residues in the N‐terminal signal sequence, which influence signal recognition particle binding and translocation dynamics^21^, ^22^; (2) co‐translational and post‐translational phosphorylation modifications that alter protein conformation and membrane insertion efficiency^23^; (3) interactions with molecular chaperones in the endoplasmic reticulum that guide proper folding and membrane integration^24^; and (4) the local lipid composition of the endoplasmic reticulum membrane at the site of insertion, which affects the energetics of transmembrane domain integration.^25^ Additionally, the distinct intracellular trafficking pathways leading to either the plasma membrane or the NSV membranes may involve cargo‐sorting machinery that selectively directs CD38 to specific membrane compartments, though the molecular determinants of this sorting process remain to be elucidated.

This configurational plasticity of CD38 gives rise to profound functional divergence with direct physiological consequences. Type II CD38 localised at the plasma membrane utilises extracellular NAD^+^ as its substrate, which is present in the interstitial space at micromolar concentrations and can be released from cells under various physiological and pathological conditions.^26^ cADPR generated by plasma membrane‐anchored CD38 must subsequently traverse the membrane to exert its intracellular actions, potentially involving yet‐unidentified transport mechanisms. In contrast, type III CD38 positioned on NSV membranes directly accesses the cytosolic NAD^+^ pool, which exists at substantially higher concentrations typically in the hundreds of micromolar range, and immediately releases cADPR into the cytosol adjacent to its target Ca^2+^‐mobilising channels. This topological configuration might maximise the coupling efficiency of NAD^+^ metabolism and Ca^2+^ signalling, particularly at hormone‐release sites, and represents an elegant example of subcellular compartmentalisation in second‐messenger signalling.^21^

The presence of type III CD38 in NSVs may also clarify previously puzzling aspects of CD38 biology. The phenotype of CD38‐deficient mice, characterised by severely impaired oxytocin release despite preserved peptide synthesis, has long suggested that CD38 contributes to neuroendocrine secretion in a manner not fully explained by models that place the enzyme exclusively at the plasma membrane.^10^ Our findings suggest that a physiologically important pool of CD38 is located on the vesicle membrane itself, thereby positioning the enzyme close to the sites where cADPR‐dependent Ca^2+^ mobilisation supports regulated exocytosis. This compartmentalised localisation provides a coherent explanation for why oxytocin release is selectively disrupted in CD38‐null mice, whereas other neuroendocrine functions appear to remain relatively intact.

## 5 CONCLUSION

This study underscores the importance of intracellular localisation and membrane topology in determining the functional properties of membrane‐associated enzymes. We show that CD38, when positioned on the NSV membranes with its catalytic domain facing the cytosol, can efficiently generate second messengers that drive the localised Ca^2+^ mobilisation required for oxytocin release. These findings suggest that the subcellular positioning of CD38 is a key determinant of its physiological function. Future investigations will be needed to assess whether comparable localisation‐dependent mechanisms operate in other secretory systems, which may prompt a broader reconsideration of exocytotic regulation in terms of intracellular enzyme compartmentalisation.

## AUTHOR CONTRIBUTIONS

T.M., A.M., and H.S. performed immunoelectron microscopy analyses. A.O. and T.O. performed Western immunoblotting and biochemical experiments. C.S. and K.M. performed cryo‐electron microscopy analyses. H.S. wrote the paper. T.M. and A.M. contributed equally to this study. H.S. supervised the whole study. All authors had full access to all study data and take responsibility for the integrity and accuracy of the data analysis.

## FUNDING INFORMATION

This study was partly supported by Grants‐in‐Aid for Scientific Research from the Japan Society for the Promotion of Science (JSPS) KAKENHI, Japan (to H.S.; 22H02656; 22K19332; to T.O.; 23K14230; 25K00107), by the Takeda Science Foundation, Japan (to H.S.; Bioscience Research Grants; to T.O.; Life Science Research Grants), by the Naito Foundation, Japan (to H.S.; Naito Memorial Grant for Natural Science Researchers), by the Ryobi Teien Memory Foundation, Japan (to T.O. and H.S.; Research Grants), by the Wesco Scientific Promotion Foundation, Japan (to T.O. and H.S.; International Travel Grants; Research Grants), and by the Japan Foundation for Applied Enzymology, Japan (to H.S.; Research Grants), Nippon Shinyaku, Japan (to T.O.; Research Grant).

## CONFLICT OF INTEREST STATEMENT

The authors declare no conflicts of interest.

## Data Availability

The data that support the findings of this study are available from the corresponding author upon reasonable request.

## References

[1] Jackson DG , Bell JI . Isolation of a cDNA encoding the human CD38 (T10) molecule, a cell surface glycoprotein with an unusual discontinuous pattern of expression during lymphocyte differentiation. J Immunol. 1990;144(7):2811‐2815. 2319135

[2] Malavasi F , Deaglio S , Funaro A , et al. Evolution and function of the ADP ribosyl cyclase/CD38 gene family in physiology and pathology. Physiol Rev. 2008;88(3):841‐886. 18626062 10.1152/physrev.00035.2007

[3] Higashida H . Somato‐axodendritic release of oxytocin into the brain due to calcium amplification is essential for social memory. J Physiol Sci. 2016;66(4):275‐282. 26586001 10.1007/s12576-015-0425-0 PMC4893072

[4] Lee HC . Cyclic ADP‐ribose and nicotinic acid adenine dinucleotide phosphate (NAADP) as messengers for calcium mobilization. J Biol Chem. 2012;287(38):31633‐31640. 22822066 10.1074/jbc.R112.349464 PMC3442497

[5] Okamoto H . The CD38‐cyclic ADP‐ribose signaling system in insulin secretion. Mol Cell Biochem. 1999;193(1–2):115‐118. 10331647

[6] Galione A . Cyclic ADP‐ribose: a new way to control calcium. Science. 1993;259(5093):325‐326. 8380506 10.1126/science.8380506

[7] Galione A , White A . Ca^2+^ release induced by cyclic ADP‐ribose. Trends Cell Biol. 1994;4(12):431‐436. 14731692 10.1016/0962-8924(94)90104-x

[8] Walum H , Young LJ . The neural mechanisms and circuitry of the pair bond. Nat Rev Neurosci. 2018;19(11):643‐654. 30301953 10.1038/s41583-018-0072-6 PMC6283620

[9] Froemke RC , Young LJ . Oxytocin, neural plasticity, and social behavior. Annu Rev Neurosci. 2021;44:359‐381. 33823654 10.1146/annurev-neuro-102320-102847 PMC8604207

[10] Jin D , Liu HX , Hirai H , et al. CD38 is critical for social behaviour by regulating oxytocin secretion. Nature. 2007;446(7131):41‐45. 17287729 10.1038/nature05526

[11] Ludwig M , Leng G . Dendritic peptide release and peptide‐dependent behaviours. Nat Rev Neurosci. 2006;7(2):126‐136. 16429122 10.1038/nrn1845

[12] Peterson MF , Otoc N , Sethi JK , Gupta A , Antes TJ . Integrated systems for exosome investigation. Methods. 2015;87:31‐45. 25916618 10.1016/j.ymeth.2015.04.015

[13] Satoh K , Oti T , Katoh A , et al. In vivo processing and release into the circulation of GFP fusion protein in arginine vasopressin enhanced GFP transgenic rats: response to osmotic stimulation. FEBS J. 2015;282(13):2488‐2499. 25846300 10.1111/febs.13291

[14] RRID:AB_3719897. http://antibodyregistry.org/AB_3719897

[15] RRID:AB_11212999. http://antibodyregistry.org/AB_11212999

[16] Martucci LL , Launay JM , Kawakami N , et al. Endolysosomal TPCs regulate social behavior by controlling oxytocin secretion. Proc Natl Acad Sci U S A. 2023;120(7):e2213682120. 36745816 10.1073/pnas.2213682120 PMC9963339

[17] Castel M , Morris JF , Whitnall MH , Sivan N . Improved visualization of the immunoreactive hypothalamo‐neurohypophysial system by use of immuno‐gold techniques. Cell Tissue Res. 1986;243(1):193‐204. 2417720 10.1007/BF00221868

[18] RRID:AB_2313960. https://scicrunch.org/resolver/AB_2313960

[19] Otubo A , Maejima S , Oti T , et al. Immunoelectron microscopic characterization of vasopressin‐producing neurons inthe hypothalamo‐pituitary axis of non‐human primates by use offormaldehyde‐fixed tissues stored at −25°C for several years. Int J Mol Sci. 2021;22(17):9180. 34502087 10.3390/ijms22179180 PMC8430530

[20] Zhao YJ , Zhang HM , Lam CMC , Hao Q , Lee HC . Cytosolic CD38 protein forms intact disulfides and is active in elevating intracellular cyclic ADP‐ribose. J Biol Chem. 2011;286(25):22170‐22177. 21524995 10.1074/jbc.M111.228379 PMC3121361

[21] Zhao YJ , Lam CM , Lee HC . The membrane‐bound enzyme CD38 exists in two opposing orientations. Sci Signal. 2012;5(241):ra67. 22969159 10.1126/scisignal.2002700

[22] Vonheijne G . The distribution of positively charged residues in bacterial inner membrane‐proteins correlates with the trans‐membrane topology. EMBO J. 1986;5(11):3021‐3027. 16453726 10.1002/j.1460-2075.1986.tb04601.x PMC1167256

[23] Goder V , Spiess M . Molecular mechanism of signal sequence orientation in the endoplasmic reticulum. EMBO J. 2003;22(14):3645‐3653. 12853479 10.1093/emboj/cdg361 PMC165631

[24] Ellgaard L , Helenius A . Quality control in the endoplasmic reticulum. Nat Rev Mol Cell Biol. 2003;4(3):181‐191. 12612637 10.1038/nrm1052

[25] Cymer F , von Heijne G , White SH . Mechanisms of integral membrane protein insertion and folding. J Mol Biol. 2015;427(5):999‐1022. 25277655 10.1016/j.jmb.2014.09.014 PMC4339636

[26] Lee HC , Zhao YJ . Resolving the topological enigma in Ca^2+^ signaling by cyclic ADP‐ribose and NAADP. J Biol Chem. 2019;294(52):19831‐19843. 31672920 10.1074/jbc.REV119.009635 PMC6937575

